# Diesel exposure suppresses natural killer cell function and resolution of eosinophil inflammation: a randomized controlled trial of exposure in allergic rhinitics

**DOI:** 10.1186/s12989-016-0135-7

**Published:** 2016-05-06

**Authors:** Erica A. Pawlak, Terry L. Noah, Haibo Zhou, Claire Chehrazi, Carole Robinette, David Diaz-Sanchez, Loretta Müller, Ilona Jaspers

**Affiliations:** 1 Center for Environmental Medicine, Asthma and Lung Biology, University of North Carolina at Chapel Hill, 104 Mason Farm Rd, Campus Box 7310, Chapel Hill, NC 27599-7310 USA; 2 Department of Pediatrics, University of North Carolina at Chapel Hill, Chapel Hill, NC USA; 3 Department of Biostatistics, University of North Carolina at Chapel Hill, Chapel Hill, NC USA; 4 Department of Medicine, University of North Carolina at Chapel Hill, Chapel Hill, NC USA; 5 United States Environmental Protection Agency, Chapel Hill, NC USA; 6 University Children’s Hospital Basel, Basel, Switzerland

**Keywords:** Natural killer cell, Diesel exhaust, Eosinophil, Resolution of inflammation

## Abstract

Exposure to diesel exhaust (DE) is known to exacerbate allergic inflammation, including virus-induced eosinophil activation in laboratory animals. We have previously shown that in human volunteers with allergic rhinitis a short-term exposure to DE prior to infection with the live attenuated influenza virus (LAIV) increases markers of allergic inflammation in the nasal mucosa. Specifically, levels of eosinophilic cationic protein (ECP) were significantly enhanced in individuals exposed to DE prior to inoculation with LAIV and this effect was maintained for at least seven days. However, this previous study was limited in its scope of nasal immune endpoints and did not explore potential mechanisms mediating the prolonged exacerbation of allergic inflammation caused by exposure to DE prior to inoculation with LAIV. In this follow-up study, the methods were modified to expand experimental endpoints and explore the potential role of NK cells. The data presented here suggest DE prolongs viral-induced eosinophil activation, which was accompanied by decreased markers of NK cell recruitment and activation. Separate in vitro studies showed that exposure to DE particles decreases the ability of NK cells to kill eosinophils. Taken together, these follow-up studies suggest that DE-induced exacerbation of allergic inflammation in the context of viral infections may be mediated by decreased activity of NK cells and their ability to clear eosinophils.

## Background

Studies in experimental animal models and controlled human exposures suggest that exposure to diesel exhaust (DE) or DE particles may act to heighten allergic respiratory inflammation and immune responses, especially in the context of viral infections [1, 2]. We have previously demonstrated that in mice sensitized to ovalbumin, exposure to DE particles prior to infection with influenza virus significantly enhances allergic inflammation, as marked by influx of eosinophils [3]. Similarly, published data from our group found that short-term exposure of human volunteers to DE (100 μg/m^3^ for 2 h) significantly increases live attenuated influenza virus (LAIV)-induced eosinophilic cationic protein (ECP) levels and virus quantity in nasal secretions of allergic rhinitic adult volunteers [4]. Analysis of ECP levels in nasal lavage is a marker of eosinophil degranulation and has been used as an indicator of nasal eosinophilia in previous clinical studies [5–7]. Interestingly, enhanced ECP levels were seen for many days after DE exposure and infection with LAIV, suggesting that the increased markers of eosinophil activation persisted after the virus had been cleared. Thus, in both mouse and human in vivo models, exposure to DE prior to infection with virus significantly increases eosinophilia and markers of eosinophilic inflammation. However, potential mechanisms mediating the enhanced and persistent activation of eosinophils in the airways after exposure to DE is not clear.

In recent years, understanding of resolution of allergic inflammation and the role of natural killer (NK) cells in these responses has increased greatly. In addition to clearing tumor and virus-infected cells, multiple recent studies have implicated NK cells in the resolution of allergic inflammation, as reviewed by Barnig and Levy [8]. Most notably, mice depleted of NK cells were found to have increased viral-induced Th2 responses in response to RSV challenge [9]. Additionally, NK cells have been shown to aggregate in the respiratory tissue draining lymph nodes in a mouse model of allergic inflammation; depletion of these cells or interference with their recruitment delayed resolution [10]. In human experiments, co-culture of NK cells with autologous eosinophils promoted apoptosis of eosinophils [11, 12], supporting a role for NK cells in resolving eosinophilia. Previous studies from our group have demonstrated a marked decrease in nasal NK cell cytokine production, cytotoxic granule production, and cell-mediated cytotoxicity when they are exposed to DE particles in vitro [13]. We therefore hypothesized that elevated and prolonged nasal ECP levels as a marker for eosinophilia in allergic subjects exposed to DE prior to inoculation with LAIV may be caused by DE-induced suppression of NK cell function. The purpose of this study was to connect persistent ECP levels in DE-exposed allergic rhinitics with impaired NK cell function in an in vivo exposure model.

## Methods

### In vivo exposure

**Fig. 1:**
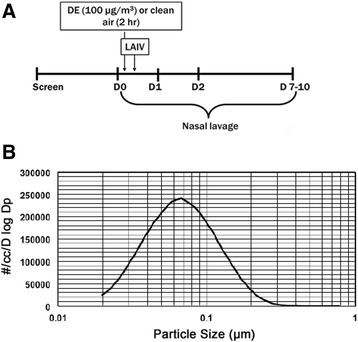
**a** Schematic of treatment protocol; **b** Representative Particle Size characteristics measured by Scanning Mobility Particle Sizer (SMPS™)

A total of 22 otherwise healthy allergic rhinitics (*n* = 11 per treatment group) were recruited and completed the study protocol depicted in Fig. 1a. Subjects were skin tested for sensitivity to a panel of common household and environmental allergens. Age, gender, and BMI did not significantly differ between the treatment groups (Table 1). Subjects were randomized to be exposed to either clean air or 110 μg/m^3^ (total particles; see Table 2) of DE for 2 h produced by a Cummins diesel generator running at 25 % load, with an organic carbon (OC):elemental carbon (EC) ratio of 0.4 for diesel particles. The detailed methods for determining OC:EC in this system have been reported elsewhere [14] and levels of gas-phase pollutants and particle characterizations are summarized in Tables 2 and 3 and Fig. 1b. Immediately following exposure to air or DE, subjects were given a standard dose of LAIV in both nostrils to simulate an influenza virus infection. Nasal lavage samples were collected prior to exposure and at days 1, 2, and 7–10 post exposure and LAIV [3]. A schematic of the experimental design is summarized in Fig. 1a. Cell free nasal lavage fluid (NLF) samples were analyzed for mediators related to eosinophil and NK cell recruitment and activation, as well as pro-inflammatory cytokines and chemokines. Immune cells isolated from the lavage were subjected to flow cytometric staining and characterization. The study was approved by the UNC Biomedical Institutional Review Board and by the U.S. Environmental Protection Agency.

**Table 1:** Demographic information for enrolled subjects

Demographic	Air (*n* = 11)	DE (*n* = 11)	*p*-value
Age (yr)^a^	27.5 ± 8.7	25.6 ± 4.7	0.52
BMI^a^	27.2 ± 5.8	25 ± 5.4	0.37
Male/female^b^	8/3	5/6	0.38

^a^t-test, ^b^Fisher’s exact test

**Table 2:** DE Particle characteristics

TEOM® (μg/m^3^ ± Std. Dev.) (min-max)	110.53 ± 0.62 μg/m^3^ (109.27–111.74)
Particle Conc. (⋕/cc ± Std. Dev.) (min-max)	236,297 ± 35,780 (197,181–297,240)
Number Mean Diameter (μm ± Std. Dev.) (min-max)	0.0375 ± 0016 μm (0.0350–0.0400)

**Table 3:** Exposure concentrations for Air and DE-exposure groups. Data are the ranges from direct measurements of DE components monitored continuously for all subjects

Group	PM	NO	NO_2_	CO	SO_2_
Air	N/A	0.00–0.01 ppm	0.00 ppm	0.00–0.07 ppm	0.001–0.004 ppm
DE	109.3–111.7 μg/m^3^	0.037–0.47 ppm	0.347–0.476 ppm	0.75–1.03 ppm	0.002–0.007 ppm

*PM* particulate matter concentration exposure concentration, *NO* nitric oxide, *NO2* nitrous oxide, *CO* carbon monoxide, *SO2* sulfur dioxide

### Collection and processing of nasal lavage fluid

Sterile saline (0.9 % NaCl w/v) was aerosolized and inhaled 5 times into one nostril and expelled into a sample cup while occluding the opposite nostril. This was repeated a total of 8 times for each side. The lavage was pipetted through a 40 μm cell strainer to remove large debris, squamous epithelial cells, and mucous, and the filtered fraction centrifuged to obtain a cell pellet and cell-free nasal lavage fluid fraction. The contents of the cell strainer were treated with a 20× dilution of Sputolysin® reagent (EMD Millipore), then pipetted through a second cell strainer, and the resulting cell pellet combined with the initial cell pellet for downstream analysis.

### Protein expression in cell-free nasal lavage fluid

Cell-free nasal lavage fluid was assayed for chemokine and pro-inflammatory cytokine expression via MesoScale Discovery® multi-spot assay system (Human Chemokine Panel 1 and Pro-Inflammatory Panel 1, respectively). Eosinophilic cationic protein, perforin, and granzyme B levels were measured via commercially available sandwich ELISA.

### Surface marker phenotyping of nasal lavage leukocytes

The cellular fraction of the nasal lavage fluid was subjected to an F(c) block, and incubated with antibodies against human CXCR3-PE-Cy5.5, CD56-PE, CD16-FITC, CD3-APC, and CD45-APC-Cy7. Cells were washed, fixed, and acquired on a BD™ LSR II flow cytometer. Compensation was performed with BD™ Anti-Mouse Ig CompBeads per manufacturer instructions, and IgG1 isotype controls were performed using autologous peripheral blood mononuclear cells from each subject.

### In vitro cytotoxicity challenge

Peripheral blood was collected from 7 healthy human subjects. NK cells enriched via density centrifugation to isolate peripheral blood mononuclear cells, and NK cells isolated from these using the Dynabeads® Untouched™ Human NK Cell negative selection kit (ThermoFisher Scientific). NK cells were incubated with vehicle (buffered saline) or DE particles obtained from exposure chamber filters, sonicated into an HBSS suspension, at a concentration of 10 μg/mL in RPMI-1640 media supplemented with 10 % fetal bovine serum overnight. Following treatment, NK cells were co-incubated with a CFSE-labeled eosinophil cell line (EOL-1) at a 3:1 NK to target cell ratio for four hours and both cell types stained with Live/Dead® Fixable Dead Cell Stain (ThermoFisher Scientific). Cells were acquired as described above.

### Statistics

Measurements for each subject were normalized to their individual baseline and expressed as fold change relative to baseline. Baseline values did not significantly differ between treatment groups (Table 4). Differences amongst exposure groups and between treatment days and baseline were compared using Mann-Whitney non-parametric t-tests via GraphPad Prism v.6. *P* < 0.05 was considered significant.

**Table 4:** Raw values (+/− standard deviation) for proteins measured at each time point

Analyte	A0	D0	*p*-value Air vs. Diesel	A1	*p*-value fold Change vs. day 0	D1	*p*-value fold Change vs. day 0	*p*-value day 1 Δ Air vs. Δ Diesel	A2	*p*-value Fold Change vs. day	D2	*p*-value Fold Change vs. day	*p*-value day 1 Δ Air vs. Δ Diesel	A7-10	*p*-value Fold Change vs. day	D7-10	*p*-value Fold Change vs. day	*p*-value day 1 Δ Air vs. Δ Diesel
ECP (pg/mL)	160.80 ± 168.02	88.60 ± 136.33	0.26	92.78 ± 111.3	0.28	112.1 ± 97.54	0.10	**0.04**	134.0 ± 177.0	0.55	98.77 ± 88.44	**0.02**	**0.02**	158.7 ± 226.9	0.56	87.99 ± 93.66	0.24	0.20
Eotaxin-1 (pg/mL)	457.94 ± 456.0	386.76 ± 190.12	0.73	498.47 ± 413.36	0.16	541.76 ± 180.13	**0.01**	0.64	541.29 ± 302.67	**0.01**	573.27 ± 164.43	**0.01**	0.20	611.63 ± 378.14	**0.09**	734.22 ± 418.35	**0.06**	0.78
IP-10 (pg/mL)	12923.64 ± 26721.96	9991.60 ± 18496.68	0.98	21627 ± 50661	0.11	6829 ± 13175	**0.02**	**0.007**	62228 ± 168311	**0.003**	18059 ± 32152	0.16	**0.08**	37629 ± 100776	**0.08**	27868 ± 64717	**0.05**	0.81
CXCR3 (MFI)	10050.0 ± 368.76	13521.22 ± 6214.61	0.40	8693.36 ± 4076.98	0.69	8551.67 ± 630921	0.44	0.15	8921.27 ± 2520.99	0.81	8604.67 ± 3702.20	0.16	0.21	9858.60 ± 2904.61	0.47	9534.75 ± 5619.12	0.13	0.53
Granzyme B (pg/mL)	59.99 ± 105.89	21.58 ± 15.63	0.21	38.26 ± 33.15	0.82	34.08 ± 30.00	**0.05**	**0.098**	50.37 ± 55.84	0.13	29.75 ± 32.93	0.17	0.92	38.19 ± 46.88	0.92	166.47 ± 393.99	**0.009**	**0.098**
Perforin (pg/mL)	161.29 ± 413.53	51.61 ± 33.61	0.39	126.65 ± 276.71	0.70	83.97 ± 97.26	0.12	0.22	123.32 ± 257.77	0.56	55.80 ± 36.13	0.21	0.47	211.79 ± 391.73	0.85	218.74 ± 429.65	0.10	0.22

*MFI* mean fluorescence intensity. *P* values based on fold change over each individual’s baseline (D0) value. *P* value between treatment groups compares protein expression relative to baseline, air exposure vs. diesel exposure. *P*-values generated via un-paired, non-parametric Mann-Whitney t-test

significant differences are in bold

## Results

**Fig. 2:**
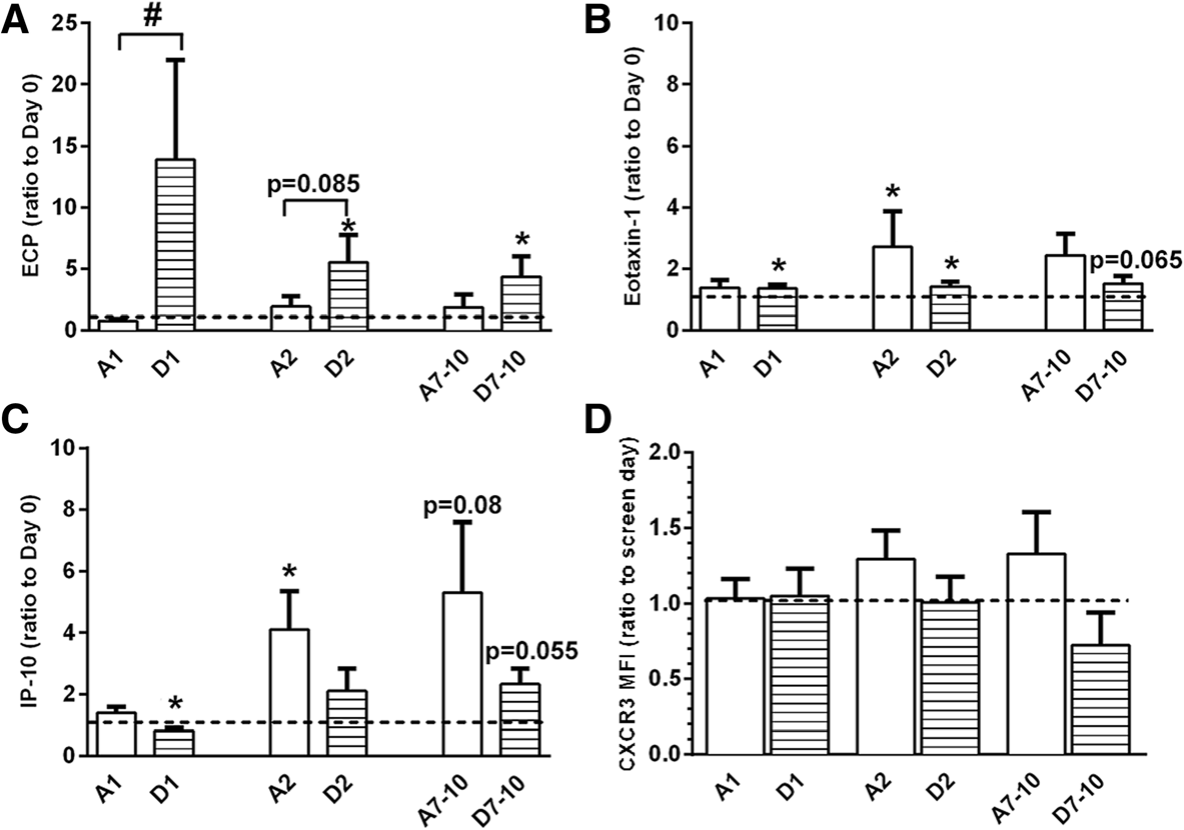
**a** protein expression of eosinophil cationic protein (ECP), **b** eotaxin-1, and **c** IP-10 in nasal lavage fluid, and **d** CXCR3 on NK cells isolated from nasal lavage. *p < 0.05 vs. baseline (day 0 or screen day), # *p* < 0.05 vs. air exposure, Mann-Whitney test. MFI = mean fluorescence intensity; A = Air exposure, D = Diesel exhaust exposure, number indicates days post-LAIV treatment

ECP levels did not differ between treatment groups at baseline (day 0) (Table 4), but subjects exposed to DE prior to inoculation with LAIV demonstrated a significant increase in ECP in the NLF from baseline at days 1, 2, and 7–10, which was not apparent in subjects exposed to air prior to inoculation with LAIV (Fig. 2a). Persistently elevated ECP suggests that eosinophils in these subjects remained within or near the nasal mucosa throughout the course of the study. There was a very small but significant increase in Eotaxin-1 in all days in DE exposed subjects and day 2 in air-exposed subjects (Fig. 2b), which may result in increased eosinophil recruitment. NLF protein analysis revealed no difference between air- and DE-exposed groups for changes in the cytokines IL-2, IL-4, IL-5, IL-10, IL-13, IFN-γ, or TNF-α, or the chemokines eotaxin-3, MCP-1, MCP-4, MDC, MIP-1β or TARC, at any of the time points (data not shown), which is consistent with the type of diesel utilized in this study and the overall dose of DEP [4, 15–18]. DE-exposed subjects demonstrated a significant reduction in the NK-recruiting and activating cytokine IP-10 at day 1 post exposure in NLF (Fig. 2c), though expression of the IP-10 receptor, CXCR3, was not significantly reduced amongst CD3^−^CD45^+^CD16^+^CD56^+^ NK cells (Fig. 2d). These differential expression patterns were not due to changes in NK cell number between treatment groups (Table 5).

**Table 5:** Phenotyping of cells in nasal lavage (+/− standard deviation) via flow cytometry

Endpoint	A0	D0	A1	D1	A2	D2	A7-10	D7-10
% Neutrophils (CD45^+^/CD56^-^/CD16^+^	54.36 ± 26.02	53.06 ± 27.49	60.49 ± 15.63	59.79 ± 19.25	58.42 ± 19.12	67.66 ± 11.41	54.62 ± 21.42	64.85 ± 17.18
% NK Cells (CD45^+^/CD56^+^/CD3^-^)	21.77 ± 12.48	24.0 ± 14.07	26 ± 7.69	23.43 ± 8.25	24.02 ± 6.84	22.66 ± 6.75	32.63 ± 18.38	23.36 ± 5.76
% CXCR3^+^ NK Cells	92.29 ± 19.47	98.99 ± 1.09	99.06 ± 0.99	94.56 ± 8.72	99.08 ± 0.82	92.94 ± 11.32	97.94 ± 3.68	88.5 ± 18.99

**Fig. 3:**
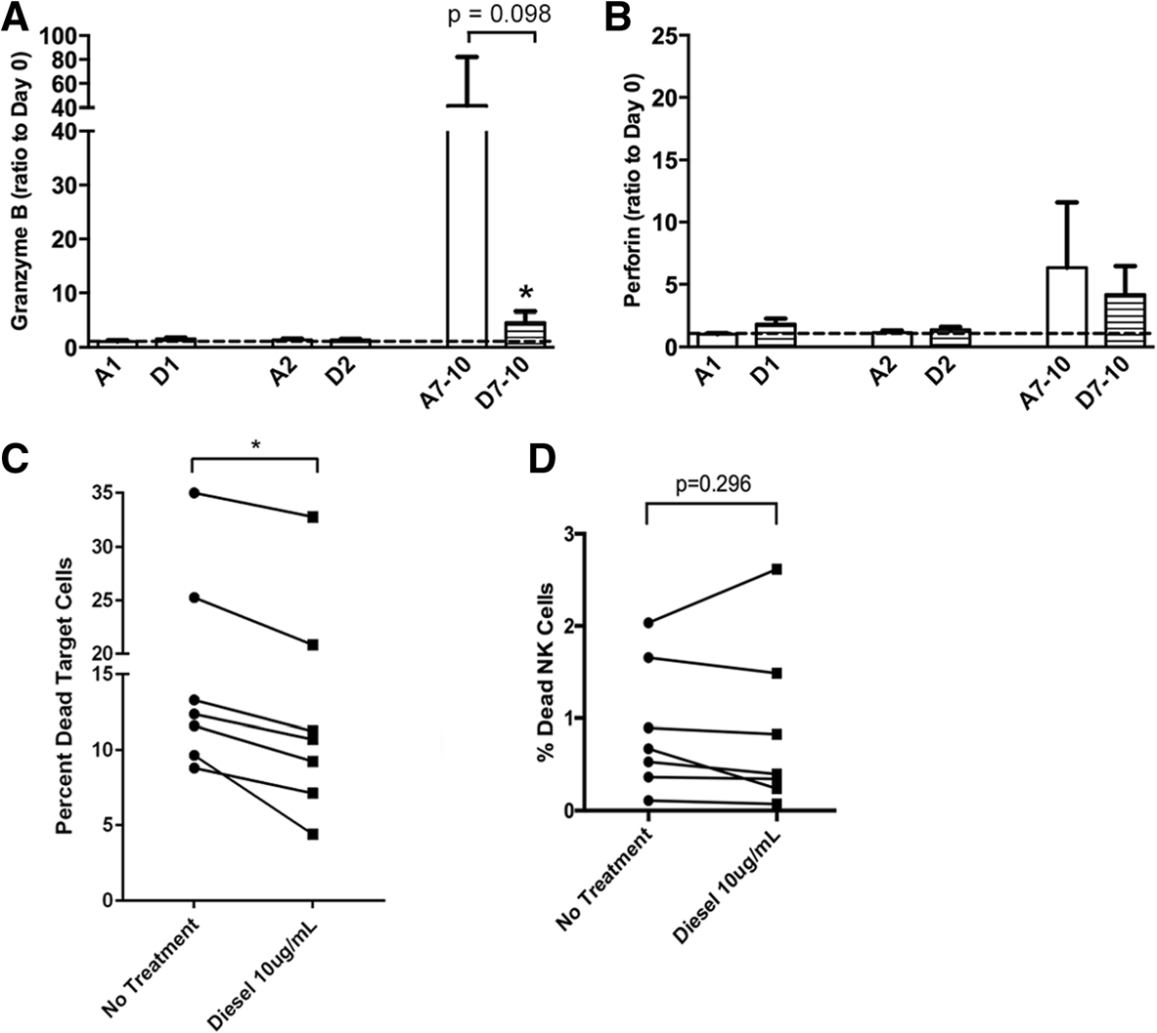
Protein expression of **a** granzyme B and **b** perforin in nasal lavage fluid. *p < 0.05 vs. air control, # *p* < 0.05 vs. baseline (day 0), Mann-Whitney test. **c** NK cell cytotoxic activity against EOL-1 target cells as determined via flow cytometry, *n* = 7 individual subjects. **d** % Dead NK cells after 24 h culture with 10 μg/mL diesel particles. * *p* < 0.05, Mann-Whitney test. A = Air exposure, D = Diesel exhaust exposure, number indicates days post-LAIV treatment

NLF samples from both treatment groups were analyzed for mediators released as part of NK cell cytotoxic granules. There was a reduction in granzyme B protein at day 7–10 in the DE exposed group (Fig. 3a) and a trend towards decreased perforin expression at the same time point (Fig. 3b). To further confirm that DE exposure impairs NK cell cytotoxic function, NK cells were isolated from peripheral blood of healthy donors and incubated with either vehicle (buffered saline) or 10 μg/mL of DE particles overnight before co-culture with an eosinophil cell line, EOL-1, at a 3:1 NK to target cell ratio. Similar to our previous studies [13], incubation of the NK cells with the EOL-1 cell line as target cells induced significant target cell killing, as determined by a fluorescent viability stain and flow cytometry, and this killing was blunted by exposure to DE particles (Fig. 3c). Loss of NK cell cytotoxic function was not due to DE treatment-induced apoptosis of NK cells (Fig. 3d). These data suggest that DE-induced suppression of NK cell function is associated with decreased ability to kill eosinophils, thus prolonging ECP persistence in the nasal lavage.

## Discussion

The data presented in this short report confirm that exposure to DE prior to inoculation with LAIV causes a significant immediate increase in ECP levels, which remained elevated as compared to baseline at least 7–10 days post-exposure and infection. While the initial increase in ECP levels may be caused by increased viral infection, our data also suggest that DE-induced reduction of NK cell killing function may be a mechanism by which DE reduces the ability to clear eosinophils and thus prolongs eosinophilic inflammation in allergic and asthmatic individuals. While previous rodent and human in vivo exposure studies have demonstrated the increased and persistent presence of eosinophils in the respiratory mucosa after exposure to DE and infection with influenza virus [2–4, 19], mechanisms mediating these responses were not clear. Data summarized in this follow-up study suggest that while the initial recruitment of eosinophils into the airways may be mediated by the effect DE has on enhancing viral infections [16, 18], the prolonged eosinophil activation seen in allergic rhinitics exposed to DE prior to inoculation with LAIV may be mediated by reducing activation of NK cells and their ability to kill eosinophils, which adds to the existing evidence implicating NK cells as active players in the resolution of eosinophilia [8].

The cellular mechanisms by which DE particles might affect NK cell function or affect NK cell-dependent clearance of eosinophils are not known. We and others have previously shown inhibitory effects of DE particles on lymphocyte cytotoxic function [13, 20]. NK cell activation depends on the expression of activating receptors, such as CD16, NKG2D, and NKp46. NK cell mediated apoptosis of neutrophils has been shown to be contact dependent and mediated by NKp46 and Fas [21], while NK cell-mediated killing of autologous eosinophils was mediated by pro-resolvin receptors [12]. We have previously demonstrated that NK cells exposed to DE particles prior to stimulation with the viral mimetic poly I:C have reduced expression of CD16 [13], a marker of overall NK cell cytotoxic activity. Thus, exposure to DE in the context of viral infection may reduce the expression of activating or pro-resolvin receptors on NK cells. There is also evidence to support a DE-induced imbalance of pro- and anti-inflammatory lipid mediators, which may preclude NK-cell granulocyte apoptosis [22]. We cannot exclude the possibility that there may be other sources of granzyme B and perforin, such as CD8^+^ T cells, however, based on our previous characterization of the cellular content of human nasal lavage [23] and the phenotyping we presented in Table 5, it is most likely that these proteins are derived from NK cell degranulation. One potential limitation of the data presented here is the inability to directly test the effects of DE exposure on nasal NK cells to induce eosinophil apoptosis in our human volunteers, since in vitro NK cell cytotoxic activity towards EOL-1 cells may involve other and/or additional mechanisms than those involved in killing eosinophils in the nasal mucosa in vivo. As a surrogate for nasal NK cell activity we assessed nasal lavage fluid levels of granzyme B, which was reduced in subjects exposed to DE. However, the much more limited time course of our ex vivo analysis on the effects of DE particles on NK cell cytotoxic function against eosinophils as compared to the reduced levels of granzyme B observed in the nasal lavage of DE-exposed subjects prohibits us to establish a direct cause-and-effect relationship in humans exposed to DE prior to inoculation with LAIV.

Despite the limitations of our small clinical cohort, and the caveats inherent in dealing with primary human samples (i.e., limitations in selecting time points, substantial dilution of protein analytes in nasal lavage, inability to measure eosinophil cell numbers in nasal lavage directly, etc.), we feel these data add significantly to our previous diesel exposure studies. The effects of DE on eosinophil-mediated inflammation have been described in guinea pigs and mice exposed to high levels of DE before [24–28]. However, our previous studies in mice demonstrated that in allergic airway disease, exposure to lower levels of DE particles alone does not enhance markers of eosinophilia, while DE prior to infection with influenza does [3, 16, 18]. In addition, recently published data exposing allergic asthmatics to DE/Air alone or with allergen challenge demonstrates that DE alone at levels similar to ours does not enhance markers of eosinophilia in allergic subjects [1, 19, 29], which is in agreement with our previous mouse study [3], and reinforces the concept that DE at these exposure levels may have little impact on its own, but a significant impact on a second stimulus. While we cannot exclude any possible direct effect of DE on ECP levels or eosinophilia in the nasal mucosa, the primary goal of this study design was to compare the effects of relevant levels of DE followed by viral infection, to air followed by viral infection in allergic rhinitics. More specifically, our aim in this study was to expand upon our earlier observation that ECP levels indeed remained elevated longer than enhanced markers of viral replication in subjects exposed to DE prior to inoculation with LAIV [4]. However, similar to our previous study a DE-alone arm was not included in the clinical study design, which precludes us from excluding the possibility that DE exposure may have a direct effect on ECP levels in subjects with allergic rhinitis.

Since experimental models suggest that DE organic carbon, specifically polycyclic aromatic hydrocarbon (PAH), may be an important variable affecting this process [15], it is important to note that we carried out the current protocol using a different diesel engine as compared to the one used in our previous study [4], which had a higher OC:EC ratio. The effects of the DE used in this study on ECP and NK cell are consistent with our previous work, however some differences were noted as well. We did not previously note a significant DE effect on IP-10, and unlike our prior study, here we did not observe a DE-associated increase IFNγ levels in the context of LAIV [3]. However, due to the limitations of the experimental conditions of the clinical study, we cannot rule out any DE-induced changes to IFNγ or any of the other measured endpoints prior to the first time point, which was approximately 24 h after the DE exposure and inoculation with LAIV. Exposure levels to the non-particulate National Ambient Air Quality Standards (NAAQS) “criteria” pollutants CO, NO_2_, and SO_2_ (Table 3) tended to be somewhat lower in the current study than in the previous study, which used emissions from an idling truck system [4]. It is possible that one or more of these factors could affect the outcomes for some of the endpoints. However, unlike the particulates, levels of these pollutants in both studies were orders of magnitude lower than the current thresholds for prevention of health effects in sensitive populations or previously reported to have significant effects on respiratory outcomes in allergic airways [30]. It is thus likely that the effects observed were due to DE particulates.

## Conclusion

While our human exposure system limits the ability to test the interaction between respiratory mucosal NK cells and eosinophils in depth mechanistically, the effects of DE particles on the ability of NK cells to kill eosinophils, even at a comparatively low dose, provides a powerful proof of concept building on ideas presented in the literature. Our data also introduces an important new clinical paradigm involving the effects of inhaled ambient air pollutants on NK cells and the reduced ability to clear eosinophils, thus exacerbating and prolonging allergic inflammation. These results are also of potential importance for public health, since exposure to particulate and traffic-related pollution, asthma, and viral infections frequently overlap, particularly in urban areas. Continuing to uncover mechanisms for how these factors interact will be critical to addressing the effects of environmental pollutants on human respiratory health.

### Declaration of ethics approval

This study was approved by the University of North Carolina Biomedical Institutional Review Board and the U.S. Environmental Protection Agency. The study was posted and maintained on clinicaltrials.gov, reference number NCT00617110.

## Abbreviations

DE: diesel exhaust
ECP: eosinophilic cationic protein
LAIV: live attenuated influenza vaccine
NK cells: natural killer cells
